# MRCNN: a deep learning model for regression of genome-wide DNA methylation

**DOI:** 10.1186/s12864-019-5488-5

**Published:** 2019-04-04

**Authors:** Qi Tian, Jianxiao Zou, Jianxiong Tang, Yuan Fang, Zhongli Yu, Shicai Fan

**Affiliations:** 1 School of Automation Engineering, University of Electronic Science and Technology of China, Chengdu, Sichuan China; 2 Center for Informational Biology, University of Electronic Science and Technology of China, Chengdu, Sichuan China

**Keywords:** Genome-wide DNA methylation, Convolutional neuro networks, Regression

## Abstract

**Background:**

Determination of genome-wide DNA methylation is significant for both basic research and drug development. As a key epigenetic modification, this biochemical process can modulate gene expression to influence the cell differentiation which can possibly lead to cancer. Due to the involuted biochemical mechanism of DNA methylation, obtaining a precise prediction is a considerably tough challenge. Existing approaches have yielded good predictions, but the methods either need to combine plenty of features and prerequisites or deal with only hypermethylation and hypomethylation.

**Results:**

In this paper, we propose a deep learning method for prediction of the genome-wide DNA methylation, in which the Methylation Regression is implemented by Convolutional Neural Networks (MRCNN). Through minimizing the continuous loss function, experiments show that our model is convergent and more precise than the state-of-art method (DeepCpG) according to results of the evaluation. MRCNN also achieves the discovery of de novo motifs by analysis of features from the training process.

**Conclusions:**

Genome-wide DNA methylation could be evaluated based on the corresponding local DNA sequences of target CpG loci. With the autonomous learning pattern of deep learning, MRCNN enables accurate predictions of genome-wide DNA methylation status without predefined features and discovers some de novo methylation-related motifs that match known motifs by extracting sequence patterns.

**Electronic supplementary material:**

The online version of this article (10.1186/s12864-019-5488-5) contains supplementary material, which is available to authorized users.

## Background

The process of DNA methylation is the selective addition of a methyl group to cytosine to form 5-cytosine under the action of DNA methyltransferase (Dnmt). DNA methylation primarily occurs symmetrically at the cytosine residues that are followed by guanine (CpG) on both DNA strands, and 70–80% of the CpG dinucleotides are methylated in the mammalian genomes [1]. The methylation status of cytosines in CpGs influences gene expression, chromatin structure and stability; and plays a vital role in the regulation of cellular processes including host defense against endogenous parasitic sequences, embryonic development, transcription, X-chromosome inactivation, and genomic imprinting, as well as possibly playing a role in learning and memory [2–5].

Determining the level of genome-wide methylation is the basis for further research. Recent technological advances have enabled DNA methylation assay and analysis at the molecular level [6–9], and high-throughput bisulfite sequencing is widely used to measure cytosine methylation at the single-base resolution in eukaryotes, including whole-genome bisulfite sequencing (WGBS) and Infinium 450 k/850 k. As the gold standard for genome-wide methylation determination, systems-level analysis of genomic methylation patterns associated with gene expression and chromatin structure can be achieved with WGBS [4, 5]. However, this method is not only expensive, but also constrained by bisulfite-converted genomes’ lower sequence complexity and reduced GC content [3]. Apart from the above issues, the unstable environment and different platforms make the situation more formidable.

Therefore, computational prediction of CpG site-specific methylation levels is critical to enable genome-wide analysis [6], and forecasting through probabilistic models and machine learning methods has already received extensive attention [7]. As has been reported, gene methylation in normal tissues is mainly concentrated in the coding region lacking CpG; conversely, although the density of CpG islands in the promoter region is high, the gene remains unmethylated. Owing to this, some typical methods focus on the predicting methylation patterns of specific genomic regions, such as CGIs [10–16]. Other methods assume that the methylation status is encoded as a binary variable, which means that a CpG site is either methylated or unmethylated [14–19]. In addition, most of the methods need to combine a large amount of information, like knowledge of predefined features [6, 11, 13–16, 18]. Considering the number of methylation sites is large (usually tens of millions), the corresponding features for prediction are not easily accessible, which leads to large amount of manual annotation and preprocessing must be implemented before obtaining the final prediction.

Here, we report MRCNN, a computational method based on convolution neural networks for prediction of genome-wide DNA methylation states at CpG-site resolution [20, 21]. MRCNN leverages associations between DNA sequence patterns and methylation levels, using 2D-array-convolution to tackle the sequence patterns and characterize the target CpG site methylation. On the one hand, MRCNN does not need any knowledge of predefined features, because it’s a deep learning method with end-to-end learning patterns. On the other hand, by using a continuous loss function to perform parameter calculations, a continuous value prediction of the methylation level can be achieved. We found that a series of convolution operations could extract DNA sequence patterns for our prediction and could yield substantially more accurate predictions of methylation from several different data sets. In addition, some de novo motifs are discovered from the filters of the convolution layer.

## Methods

### Data and encoding

We downloaded the whole genome bisulfite sequencing (WGBS) data (GEO, GSM432685) of H1 ESC from the GEO database for training and validation. The methylation level of each CpG locus is represented as a methylation ratio, varying from 0 to 1. The ratio is used as the network prediction target value, while the weights between the nodes in the network are optimized by minimizing the error between the predicted value and the target value. For independent testing, we chose genome-wide methylation data from multiple series of GEO databases, including the same series of H1 ESC (GEO, GSM432686) and different series of brain white matter, lung tissue, and colon tissue datasets (GEO, GSE52271). The DNA sequences selected were from the UCSC hg19 file, GRCh37 (Genome Reference Consortium Human Reference 37) with GenBank assembly accession number GCA_000001405.1.

In contrast to other traditional prediction tools with predefined features, our method exclusively takes the raw sequence as input. Given a DNA sequence, a fragment of 400 bps centered at the assayed methylation site was extracted. We choose the window size of 400 (without counting the target site and including each 200 bps DNA fragment upstream and downstream), with consideration for the potential workload of the calculation. Prior to conducting MRCNN training, these fragments needed to be encoded to convert the bases A, T, C, and G in the original sequence into matrices that could be input to the network. The strategy we select was one-hot encoding with the following rules: A = [0,0,0,1]; T = [1, 0, 0, 0]; C = [0, 1, 0, 0] and G = [0, 0, 1, 0]. After preprocessing, a matrix of 400*4 size could be generated for each target CpG site, in which every row represented a base (A, T, C, G) and the columns assembled the whole original fragment.

### MRCNN

**Fig. 1:**
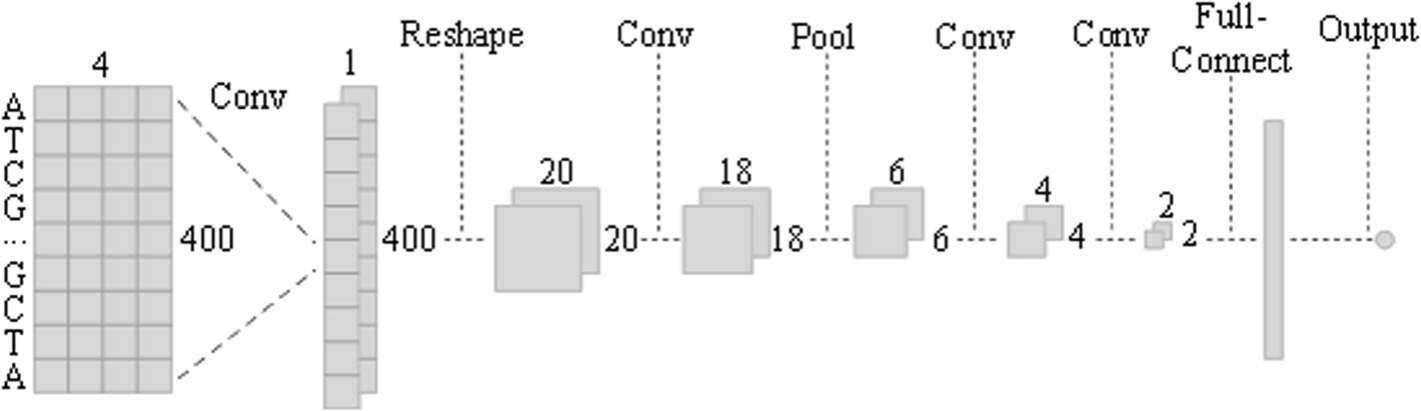
The deep-learning architecture of MRCNN. The input layer is a matrix of one-hot coding for the DNA fragment centered at the methylation site, and the first convolution layer helps extract the information of each base. Then, it is reshaped as a 2D tensor for the following operations, and the convolution and pooling operations obtain higher-level sequence feature, while the next two convolution layers overcome the side effects of the saturated zone. Finally, the tensor is expanded by the full-connection layer, and the output node gives the prediction value

Deep learning is widely used in the field of image recognition due to its end-to-end mode, by which the convolutional neural network achieves good results with its specific partial connection. However, there is a lack of knowledge on how to construct a deep learning model that could be applied to the regression of methylation levels. As we know, a typical convolutional network is generally a convolution layer adjacent to a pooling layer, alternating in turn and finally output by a fully connected layer, such as VGG Net [22]. We were more concerned about solving the regression problem itself, and after tried many structures, we eventually found that, for the prediction of methylation sites, the required structure has its own unique characteristics. On the one hand, we must consider the complete coding information of single base. On the other hand, the method needs to implement efficient feature extraction to improve the prediction results. The final deep learning architecture of MRCNN is shown in Fig. 1.

The first layer of the MRCNN is a single convolutional layer, which is mainly employed to extract single nitrogenous base information from the 400*4 input matrix. Because each base is a 1*4 independent code, the size of the convolution kernel can only be 1*4. This makes it possible to ensure that every base’s information is entered into the network while the 16 feature maps are generated. In the design of the first layer, we choose not to adopt the pooling operation because the convolution of the first layer was essentially the synthesis of coding information, that is, ensuring each base’s encoded information could be read completely by the network. For the input matrix *s*_*n*, *x*, *y*_,

$$ {L}_{n,1}=\sum \limits_{x=1}^{400}\ \sum \limits_{y=1}^4{s}_{n,x,y}{w}_{x,y}^{f,1}+{b}^{f,1} $$

Here, ${w}_{x,y}^{f,1}$ is the parameter or weight of the convolutional filter f for this layer, and *b*^*f*, 1^ is the corresponding bias. Then, the output of the first layer *L*_*n*, 1_ for each CpG site is a 400*1 tensor with 16 channels. To extract the information contained in the DNA sequence pattern, the output tensor is reshaped into a 20*20 tensor before being input into the next layer, which is advantageous for subsequent 2D-array-convolution and pooling operations. Here, each row of tensor *L*_*n*, 1_ represents the synthesis information of every single base, then it is restructured following the original queue of bases while the shape is changed to 20*20.

The second and third layer are the traditional convolution and pooling layers. The size of the convolution kernel is 3*3, the pooling method is max pooling, and the step sizes are 1*1 and 3*3. Through this layer, higher-level sequence features can be extracted.

$$ {\displaystyle \begin{array}{c}{L}_{n,2}= Relu\left(\sum \limits_{x=1}^{20}\ \sum \limits_{y=1}^{20}{L}_{n,1}{w}_{x,y}^{f,2}+{b}^{f,2}\right)\\ {}{L}_{n,3}={\mathit{\max}}_{3i\le x,3i\le y}\left({L}_{i,n,2}\right)\end{array}} $$

The Relu activation function sets negative values to zero, such that *L*_*n*, 2_ corresponds to the evidence that the motif represented by ${w}_{x,y}^{f,2}$ occurs at the corresponding position. Nonoverlapping pooling is implemented to decrease the dimensions of the input tensor and, hence, the number of model parameters.

The next two layers are both single-convolution layers with the same size and step size as the second layer’s convolution kernel. The convolution of the first layer and these two layers is linear convolution operation, with no pooling layer connection or activation function. The main purpose is to improve the effect of the convolution and nonlinear activation function, which results in part of the input falling into the saturated zone, with corresponding weights not being able to be updated. Finally, the tensor obtained by the last layer is expanded through the fully connected layer. A drop-out function is introduced for possible overfitting in training and then the methylation level could be obtained via the output layer. For the loss function in the training process, we chose the Mean Square Error (MSE) function for measurement, which is a classic solution to the problem of regression:

$$ \mathrm{MSE}\left(Y,{Y}^0\right)=\frac{\sum_{i=1}^n{\left(Y-{Y}^0\right)}^2}{n} $$

where Y represents the predicted value of methylation and *Y*^0^represents the true methylation level. Since the final predicted value is continuous, it may be more than 1 or less than 0, and we have incorporated this uniformly. For a prediction value greater than 1, the value is taken as 1, and a prediction value less than 0 is taken as 0.

### Model construction and evaluation

For all training processes and evaluations, we used a holdout validation. First, for construction of the model, we selected nearly 10 million sites from WGBS for training. Since all chromosome numbers are disrupted, it is not necessary to consider the difference among different chromosomes, which is more conducive to the discovery of the genome-wide DNA methylation patterns. Approximately 2 million CpG sites were randomly selected from the remaining sites as the validation set to help the network fine-tune the parameters. For testing the model, we randomly divided the sites in the test data set into a few copies to generate multiple independent test subsets. The division of the test set was based on two aspects, one being the original methylation level and the other being whether the region where the site is located belonged to the CpG islands. Details will be explained in the Results section. This also helps reduce the accidental errors in the model testing process, which is equivalent to a number of completely different test sets, as the training and test sites are completely different in origin. In general, we fitted the model on the training set, optimized the hyperparameters on the validation set, and performed the final model evaluation and comparison on the test sets.

To illustrate the model performance, we compared MRCNN with DeepCpG [7]. DeepCpG is the most state-of-art tool for genome-wide hypermethylation and hypomethylation prediction using deep learning. With a modular design, it uses a one-dimensional convolution DNA module and a bidirectional gated recurrent network of CpG module to achieve prediction. In addition, to compare the effect of network structural difference on the results, we also trained a simple CNN network as a baseline method. The specific structure of this network was an input layer, convolution layer 1, pooling layer 1, convolution layer 2, pooling layer 2, a fully connected layer, and an output layer. For simple CNN, we chose the same loss function and activation function to ensure univariate element during the experiments.

On the basis of the above, in order to analyze the sequence features extracted during the training of the model, we visualized the weight matrix of the convolutional filters by reverse decoding from weight assignment and corresponding raw tensor input. Specifically, the products of the first convolutional layer shared four types of weights, which corresponded to the original encoding of the four bases, so that the base sequence could be assigned according to the input, and then the weights of the different sequences could be reassigned according to the size of the filter weights. Motifs could be generated from MEME 5.0.1 by inputting the weighted sequences [23], and these de novo motifs were matched to annotated motifs given by Tomtom 5.0.1 [24]. Matches, where an FDR less than 0.05 was considered significant. All training and testing were implemented on our server with 128 G memory and 2 Nvidia 1080 graphics cards.

### Evaluation metrics

We quantitatively evaluated the predictive performance from regression and classification. For regression, we chose the root mean square error (RMSE) and mean absolute error (MAE),

$$ {\displaystyle \begin{array}{c}\mathrm{RMSE}\left(Y,{Y}^0\right)=\sqrt{\frac{\sum_{i=1}^n{\left(Y-{Y}^0\right)}^2}{n}}\\ {}\mathrm{MAE}\left(Y,{Y}^0\right)=\frac{1}{n}\sum \limits_{i=1}^n\left|Y-{Y}^0\right|\end{array}} $$

where Y represents the predicted value of the methylation level and *Y*^0^represents the true value.

For classification evaluation, we chose the sensitivity (SE), specificity (SP), classification accuracy (ACC) and area under the receiver operating characteristic curve (AUC). Here, TN, TP, FN and FP represented the number of true-negatives, true-positives, false-negatives and false-positives, respectively.

$$ {\displaystyle \begin{array}{c}\mathrm{SE}=\frac{TP}{TP+ FN}\ \mathrm{SP}=\frac{TN}{TN+ FP}\\ {}\mathrm{ACC}=\frac{TP+ TN}{TP+ FN+ TN+ FP}\end{array}} $$

## Results

To evaluate the model prediction performance, we considered the two aspects, consisted of regression errors and binary classification performance. For regression errors, the model predictions of hypermethylation, hypomethylation and intermediate methylation status were compared to analyze the predictive properties of MRCNN for CpG methylation regression. These three states were grouped by different cutoff values of the methylation rate. Analysis of the classification performance was implemented by comparing the classification metrics of sites from the CpG islands and non-CpG islands among different models, which could be more comprehensive because of the difference in methylation patterns on distinct regions of the genome. Predictions results from other tissues were used to further analyze the robustness of MRCNN for more complicated methylation mechanisms. In addition, we also analyzed the filters from the model training process, and verified the validity of the sequence feature extraction, and obtained related de novo motifs.

### Regression error

Here, to demonstrate the predictive ability for different methylation states, we distinguished successive methylation values in the raw data by different cutoff values. Most of the previous studies were focus on predictions of hypermethylation and hypomethylation, thus we also evaluated model performance based on predictions of the two states. However, in addition to this, in order to objectively evaluate the regression prediction, we added the evaluation for prediction of the intermediate methylation status. Specifically, if the original methylation label value was greater than 0.9, it was classified as “hyper”, and if it was less than 0.1, it was classified as “hypo”. The intermediate methylation status expressed as “mid” was defined by an original value greater than 0.4 but less than 0.6. Three different groups were formed and then regression results were evaluated by calculating the errors between the true and predicted values.

**Fig. 2:**
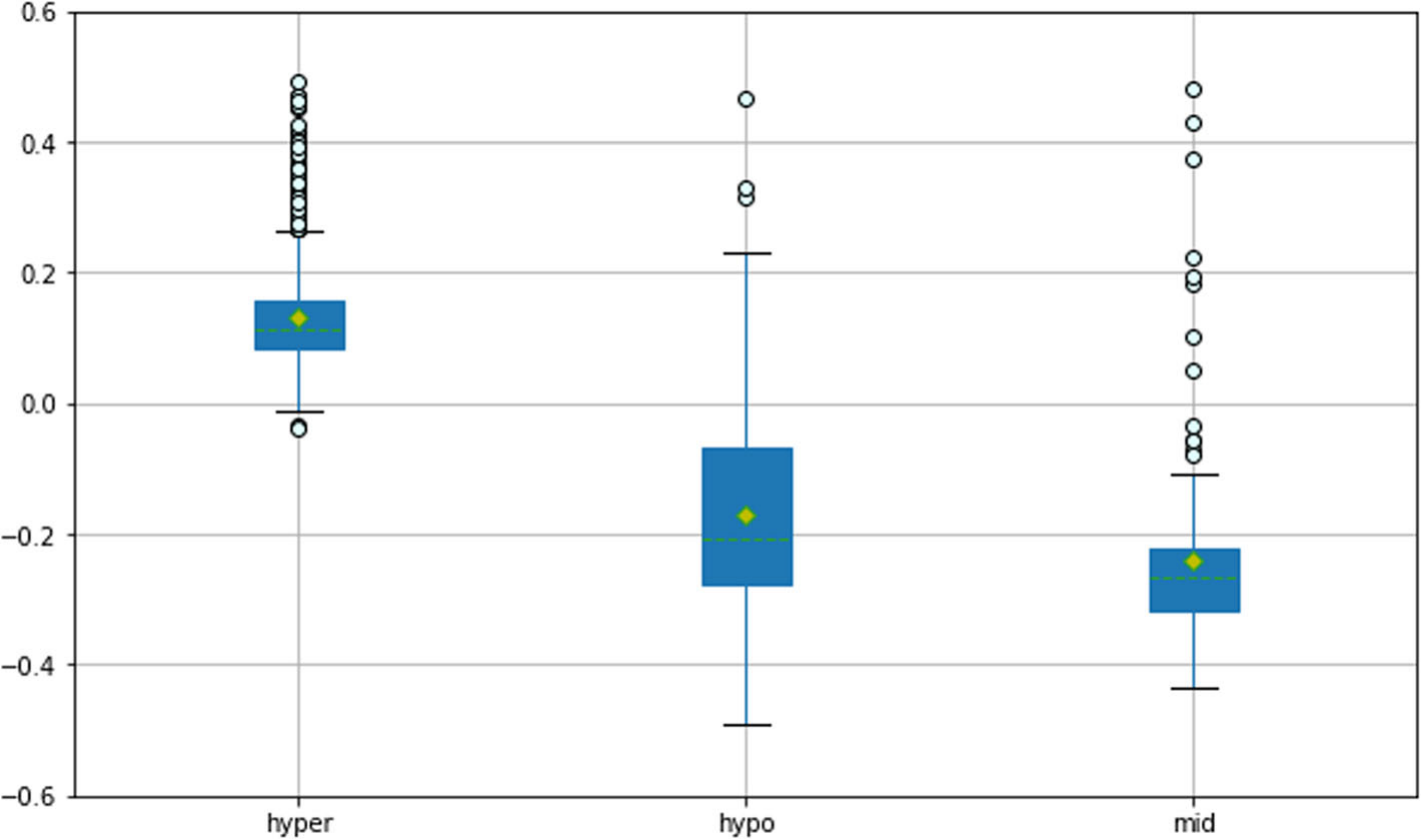
MRCNN achieved regression of the whole genome methylation. The box diagrams depict the distribution of the prediction errors of the three groups of sites. The yellow diamonds represent the mean points and the green dotted lines represent the median lines. The points outside the upper and lower boundary lines are the outliers

The different regression results of the three groups confirmed our previous expectation that MRCNN plays different roles in learning hypermethylation (hyper), hypomethylation (hypo) and intermediate methylation (mid) statuses. A comparison can be concluded from the boxplot in Fig. 2. For sites with significantly high methylation status, MRCNN was able to achieve smaller errors and obtain more satisfactory predictions compared with hypo and mid groups. On one hand, there were more sites with hypermethylation on genomes during training, on the other hand, potential more complex methylation mechanisms made prediction of hypo and mid methylation more difficult. In terms of the overall regression results, MRCNN achieved good results. First, maximum error for a single site prediction was approximately 0.5, and the prediction error distribution showed high accuracy of the predictions as most of the errors were concentrated around 0.1 for all test sites, see in Additional file 1. The RMSE and MAE of the three groups were calculated as follows: hyper: RMSE = 0.146806, MAE = 0.129885; hypo: RMSE = 0.23837, MAE = 0.207714; mid: RMSE = 0.281514, MAE = 0.268643. As seen from the RMSE and MAE values, the overall error was acceptable and would not produce a case in which a hyper site was predicted to be hypo, a hyper site was predicted to be mid, etc.

### Classification performance

Considering that most previous studies on methylation were based on CpG islands [4], the evaluation of the classification performance was implemented for loci from CpG islands and non-CpG islands. Additionally, we compared MRCNN to DeepCpG for analysis of the classification ability for methylation under different deep-learning architectures and brought in the simple CNN model as the baseline method.

**Fig. 3:**
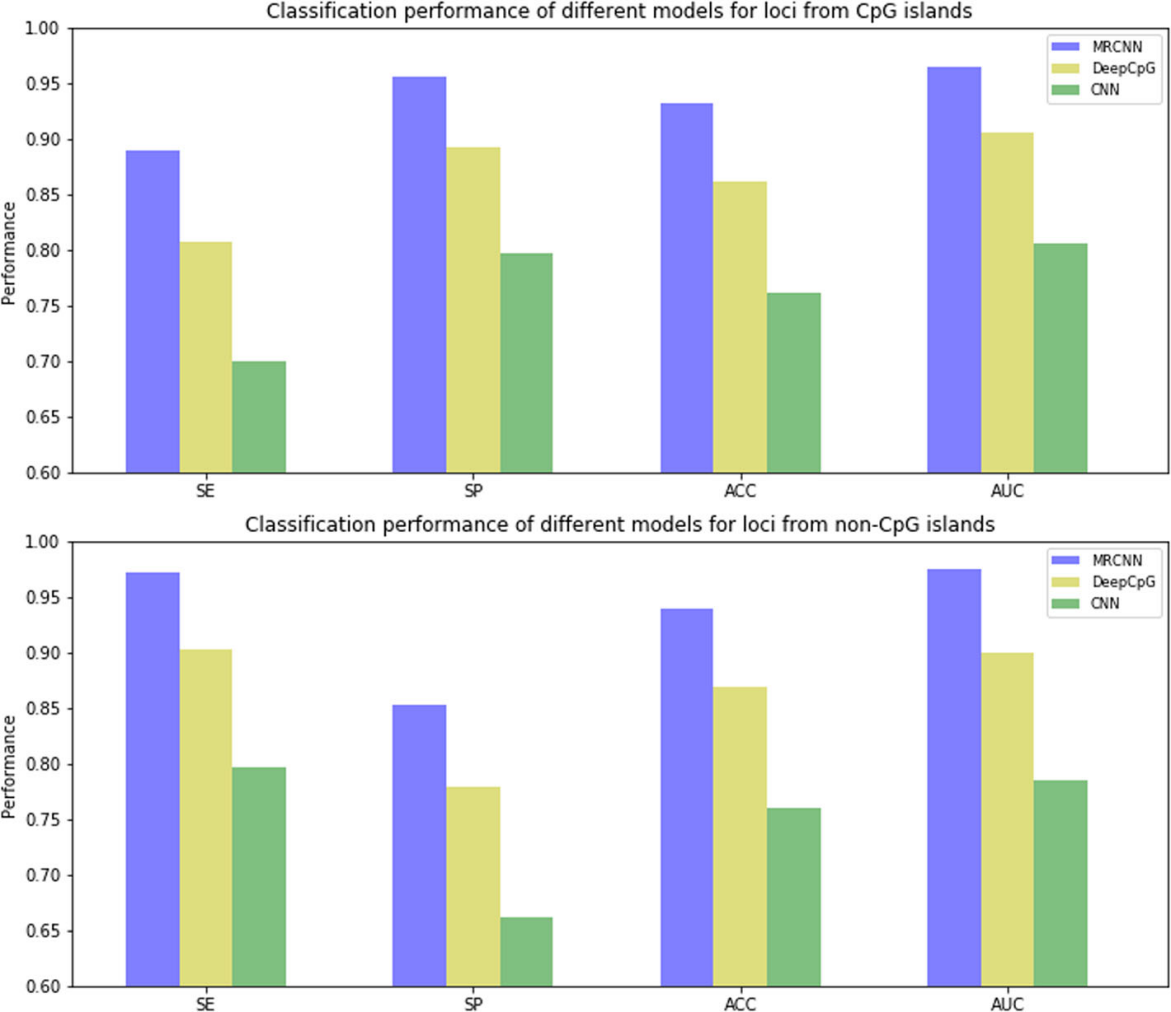
MRCNN obtained better classification performance than DeepCpG and the baseline method, simple CNN. Different deep learning architectures lead to different effects in extracting features, which in turn affects the classification results for the test sets. The difference between the SE and SP between CpG islands and non-CpG islands reveals distinct methylation patterns in different regions of the genomes

Since our label values and prediction results were continuous, we selected 0.5 as the cutoff value to divide the state of methylation into positive (> 0.5) and negative (≤0.5) samples. Via holdout validation (“Methods”), all methods were trained and tested on distinct methylation sites. In particular, these sites were previously grouped, with part of them from CpG islands and the rest from non-CpG islands. CpG islands are short CpG-rich regions of DNA which are often associated with the transcription start sites of genes. There are differences in methylation patterns between CpG islands and non-CpG islands, so we chose SE, SP, ACC and AUC to quantify the prediction performance of different models. The results of the classification comparison were shown in Fig. 3. The results showed that the overall prediction of MRCNN was better than that of DeepCpG, while the result of DeepCpG was better than that of the baseline model, CNN. It is worth mentioning that MRCNN achieved an accuracy of 93.2% and an AUC of 0.96 (t-test; *P*-value = 3.27 × 10^− 19^) on sites from CpG islands and an accuracy of 93.8% and an AUC of 0.97 (t-test; P-value = 2.65 × 10^− 19^) on sites from non-CpG islands. To fully compare the classification performance of the three models, we also selected several sets of loci from the whole genome with different sizes for testing. The results were shown in Additional file 2.

We can see that even a general simple CNN model had a certain ability to describe the relationship between DNA sequences and CG sites after training and achieved an accuracy of more than 70% and an AUC of approximately 80%. However, there was still a gap compared to the well-designed MRCNN and DeepCpG. On one hand, we can see the powerful feature extraction capability of deep convolutional networks. On the other hand, we can conclude that a customized deep learning model for a specific problem is able to truly utilize its capability. In addition, we also find that in the prediction of sites from CpG islands, the SE is less than the SP, while this situation is exactly the opposite for sites from non-CpG islands. A significant reason for this is that CpG islands are enriched with sites of hypomethylation (more negative samples), while non-CpG islands are predominantly hypermethylated (more positive samples). This illustrates the effect of the different methylation patterns of CpG islands and non-CpG islands on feature extraction during model training.

**Fig. 4:**
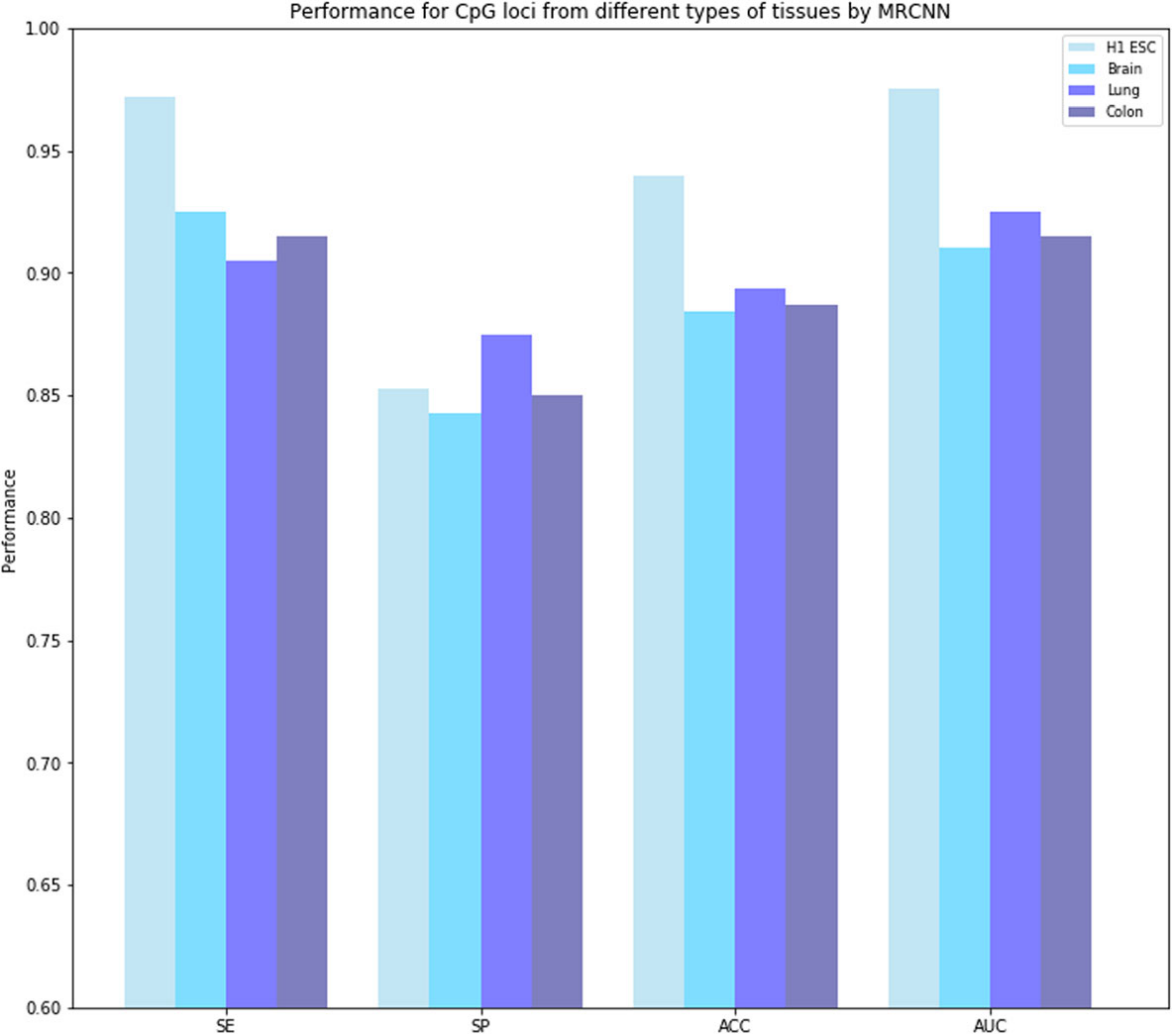
MRCNN predicted methylation for different types of tissues. The H1 ESC was used as the control data, and the other three data were taken from the normal brain white matter, lung and colon tissue. Although MRCNN was trained on H1 ESC data, it still obtained high accuracy and performance when used to predict methylation levels of other types of tissues. The results showed that MRCNN had a certain robustness to more complicated methylation problems

We also considered the effect of different cell and tissue types on the prediction of MRCNN. Based on this, test was performed on several other tissue types of methylation data. Since the data for training the model come from the normal stem cells of human body, we compared the performance of predicting the methylation level of another three tissues. The test loci come from normal brain white matter, lung tissue, and colon tissue, which were randomly distributed on CpG islands and non-CpG islands for the consideration of genome-wide methylation prediction. The results of the classification performances were shown in Fig. 4. Precisely speaking, the prediction result from the H1 ESC was slightly better than the other three cell types, but the difference was very tiny, and the prediction of hypomethylation in lung tissue was better than that of H1 ESC (with higher SP). MRCNN got an AUC of 0.91 (t-test; *P*-value = 1.87 × 10–19) for brain white matter data, an AUC of 0.925 (t-test; P-value = 2.21 × 10–19) for normal lung tissue data and an AUC of 0.915 (t-test; P-value = 4.19 × 10–19) for normal colon tissue data.

Although MRCNN was trained based on human stem cells, we can see from the experimental results that the performance of MRCNN was still good on other tissue methylation data and further demonstrated the effectiveness of MRCNN as a universal predictive tool for genome-wide methylation. For more cautious consideration, we also evaluated the prediction of MRCNN in the cancerous phenotypes of the three tissues, and the results were shown in Additional file 3. Overall, MRCNN achieved satisfactory predictions for different types of cells and tissues, indicating that the model had considerable adaptability in face of more complex methylation mechanisms and confirmed the original intention of designing a universal genome-wide methylation prediction tool.

### Feature analysis and motifs finding

**Fig. 5:**
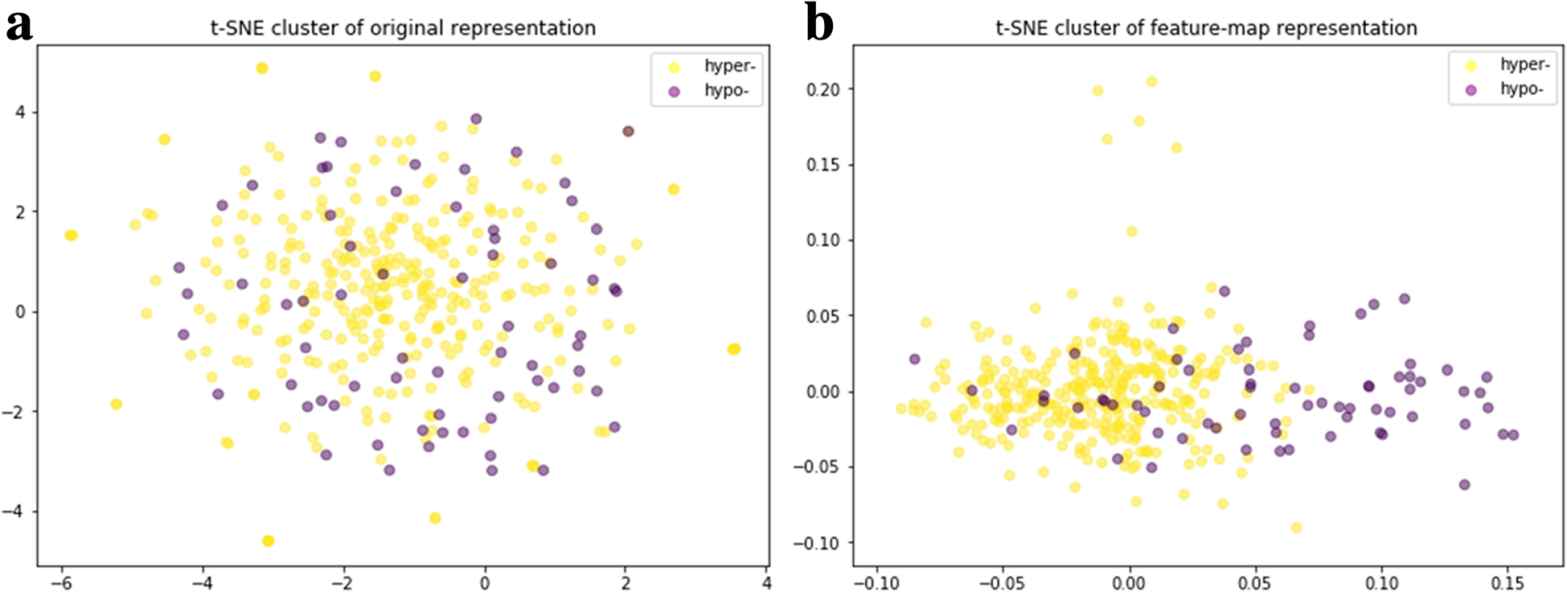
Clustering results for hypermethylation and hypomethylation loci of the original features and the learned filters of the first convolutional layer. **a** t-SNE plot of the original input tensor representation. From the plot, we cannot apparently distinguish between hypermethylation and hypomethylation. **b** t-SNE plot of the learned feature map representation. Hypermethylation and hypomethylation are generally grouped

To explore the extraction of DNA sequence pattern information during the training process, we also analyzed the feature maps from the network. In particular, we analyzed the learned filters of the first convolutional layer. First, we evaluated the ability of these filters to distinguish between hyper and hypo methylation states by visualizing the generated representations with t-SNE [25]. We compared the representation of the learned filters with the original input tensor representation and found that the learned filters were more able to distinguish the methylation level of the sites and explain the feature extraction by MRCNN. The t-SNE plot was shown in Fig. 5. The original feature could not distinguish the hyper and hypo methylation states quite well, while after the convolutional feature extraction, it could be roughly separated and would be sufficient to demonstrate the validity of the convolution operation. So, we can infer that the feature extraction was finished during the training and thus produced good prediction result.

**Fig. 6:**
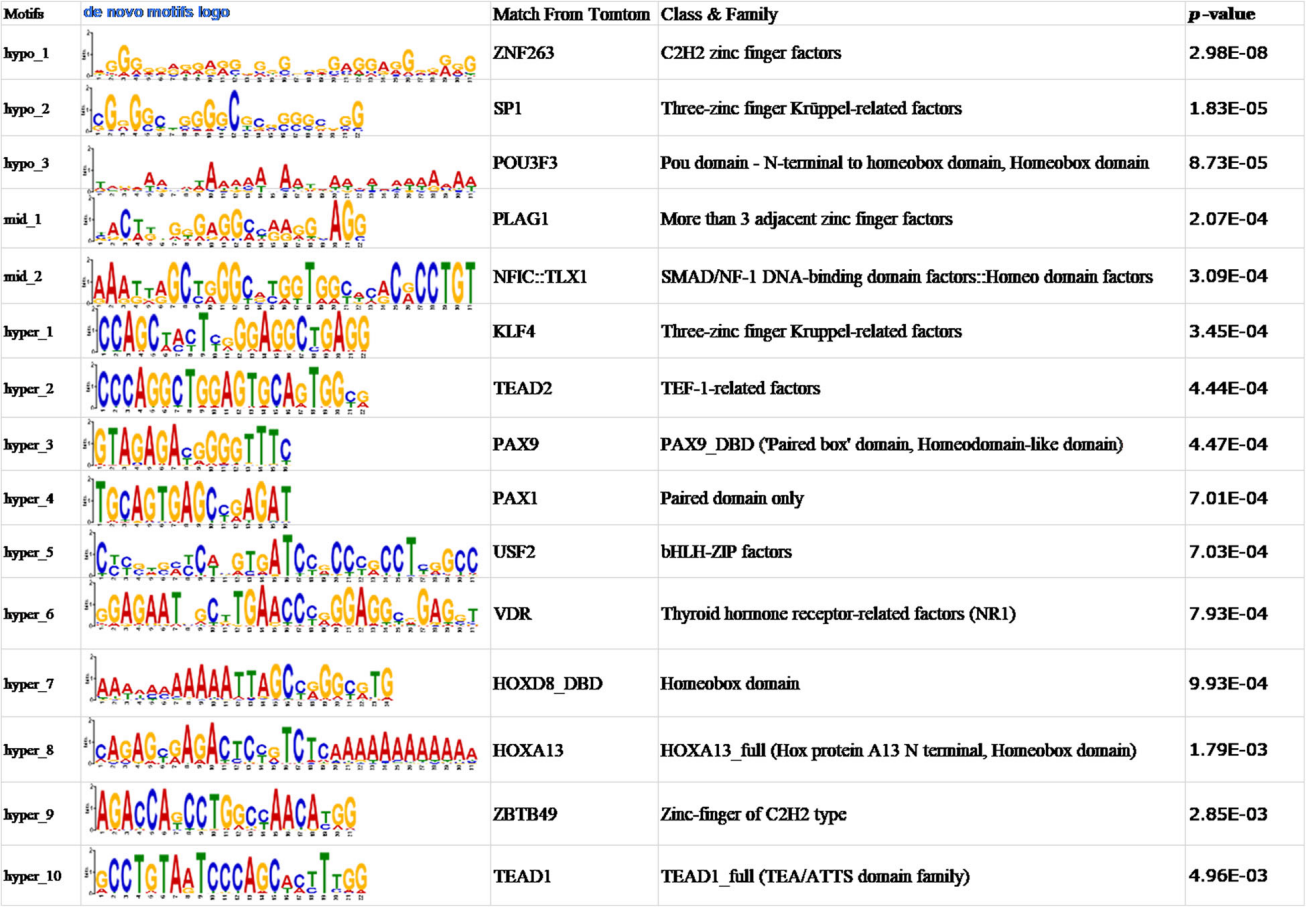
Discovered sequence motifs associated with DNA methylation. The first column is the number of de novo motifs and the second column is the motif logo generated by MEME. The third column is the known motifs matched by Tomtom, and the next forth column is the corresponding class and family representing the biological factor species of the known motifs. The *p*-value is defined as the probability that a random motif of the same width as the target would have an optimal alignment with a match score as good as or better than the target’s. Tomtom estimates the p-value using a null model consisting of sampling motif columns from all the columns in the set of target motifs

These filters also recognize DNA sequence motifs similarly to conventional position weight matrices and can be visualized as sequence logos [7]. The discovered sequence motifs associated with DNA methylation are from the online motif-based sequence analysis tools MEME [23] (version 5.0.1). We submitted these de novo motifs into Tomtom [24] (version 5.0.1) to find similar known DNA motifs by searching public databases. This may contribute to our deeper knowledge of methylation and DNA sequences. Part of the motifs and their matches were shown in the Fig. 6. The top three motifs were from hypomethylation related sequences (with methylation rate < 0.1), the middle two motifs were from sequences with a methylation rate between 0.4 and 0.6, and the last ten motifs were from hypermethylation related sequences (with methylation rate > 0.9). It was interesting that, as intuitively seen from the logo, the hypermethylated corresponding motif tended to have major bases of one certain type at a specific site, while there was no particularly obvious trend in the motif corresponding to the hypomethylation and intermediate methylation status. In addition, regardless of hypermethylation or hypomethylation sites, several of the matched known motifs were related to zinc finger factors, suggesting that it might play an important role in the methylation process. There have been reports in the literature that methylation is associated with zinc finger factors [26]. In this research, Carvin et al. pointed out that the selective targeting of methylation by zinc-finger proteins demonstrated that binding of distinct classes of factors could be monitored in living cells. Other matched motifs indicated more potential methylation background knowledge in different biological mechanisms.

## Discussion

Through multilayer convolution learning, MRCNN achieves methylation prediction for CpG loci at single-base resolution, and thanks to the continuous MSE loss function, the method enables continuous value regression. Although the 2D-convolution approach can exploit more comprehensive DNA sequence features for methylation prediction, one potential problem is that when the sequence is fixed, the predicted results do not change. Thus, we not only tested the model on the same series of tissues, but also on different types of tissues. The results showed that the impact of this problem was tiny, and the prediction results did not produce obvious fluctuations. The most important point is that MRCNN achieves methylation prediction of CpG loci by local DNA sequences, not only does it overcome the cumbersome pre-processing, but the prediction results cover all ranges of methylation values. In addition, we also find the corresponding de novo motifs through the DNA sequence pattern extracted during the training process. The significance of MRCNN is more focused on the realization of a universal model for predicting the genome-wide methylation level of sites by the local DNA sequences. We can use deep learning to fit any problem we care about through large-scale data training, but more methods and attempts would be needed for problems with complex mechanisms and broad backgrounds, such as methylation. Here, MRCNN, as a general genome-wide methylation prediction tool, does not solve all methylation-related problems, it has its own scope of application, but builds a bridge between DNA sequences and methylation at CpG loci.

The application of deep learning methods in bioinformatics has become a hot phenomenon, especially in exploring the implicit relationships and nonlinear mapping of large-scale data. Nowadays, it is possible to analyze methylation at all levels with the massive data generated by high-throughput detection technology. Thus, the combination of the two aspects gives us an opportunity to further explore the methylation mechanism. Although we can analyze the phenomenon of methylation through deep learning, we still need to understand the essence of methylation, and only by making full use of the known information can we discover new knowledge. One of the future efforts is to combine deep learning with existing known methylated biological backgrounds to construct a comprehensive analytical model to achieve deeper understanding of this epigenetic phenomenon. In addition, the learned features during the training process of MRCNN may reflect the normal and abnormal patterns of methylation, which are worthy of further study. This research will be combined with cancer data for analysis in subsequent work, which will be a key point for us to expand the model.

## Conclusion

In this paper, we propose a novel deep learning model based on convolutional neural networks for predicting DNA methylation at single-CpG-site precision using local DNA sequence. The specially designed network structure makes it a universal model for predicting genome-wide methylation of CpG loci. The extraction of DNA sequence features is achieved by multistep 2D-array-convolution, and the MSE loss function is minimized to achieve regression of the methylation values. Based on extensive training data, MRCNN achieves accurate methylation predictions and exhibits stability in prediction methylation of different types of tissues. We also further demonstrate the discovery of de novo motifs by analyzing the learned filters of the convolutional layer, and some of these motifs have been reported playing an important role in the regulation of methylation.

## Additional file

Additional file 1: Additional figures. Distribution of the differences between the predicted value and the true value of all sites. (PDF 62 kb)

Additional file 2: Additional figures. Comparsion of the comprehensive classification performance metrics including ACC and AUC on the different size of test subsets. (PDF 64 kb)

Additional file 3: Additional figures. Comparison of the classification performances in three cancerous tissues. (PDF 56 kb)

## Abbreviations

CNN: Convolutional Neural Network
GEO: gene expression omnibus
NCBI: National Center for Biotechnology Information
WGBS: whole genome bisulfite sequencing
