# Supplementary material for: MRCNN: a deep learning model for regression of genome-wide DNA methylation
Source: BMC Genomics. 2019 Apr 4;20(Suppl 2):192. doi: 10.1186/s12864-019-5488-5 (PMC6457069; doi:10.1186/s12864-019-5488-5)

Additional file 1. Distribution of the differences between the predicted value and the true value of all sites.

The sites consist of hyper, hypo and mid groups. The distribution of the overall error is mainly concentrated around 0.1, indicating that MRCNN gives a precise regression of genome-wide methylation.

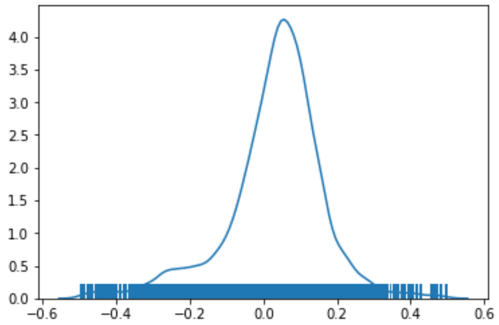

Supplement: Supplementary file 1 — Additional figures. Distribution of the differences between the predicted value and the true value of all sites. (PDF 62 kb) [file 12864_2019_5488_MOESM1_ESM.pdf]
