# Supplementary material for: MRCNN: a deep learning model for regression of genome-wide DNA methylation
Source: BMC Genomics. 2019 Apr 4;20(Suppl 2):192. doi: 10.1186/s12864-019-5488-5 (PMC6457069; doi:10.1186/s12864-019-5488-5)

Additional file 2. Comparison of the comprehensive classification performance metrics including ACC and AUC on different size of test subsets.

The three models were tested using different groups of test sets. Each group contained 5 subsets with 1000,2000,3000,4000, and 5000 CpG loci from the whole genomes, respectively.

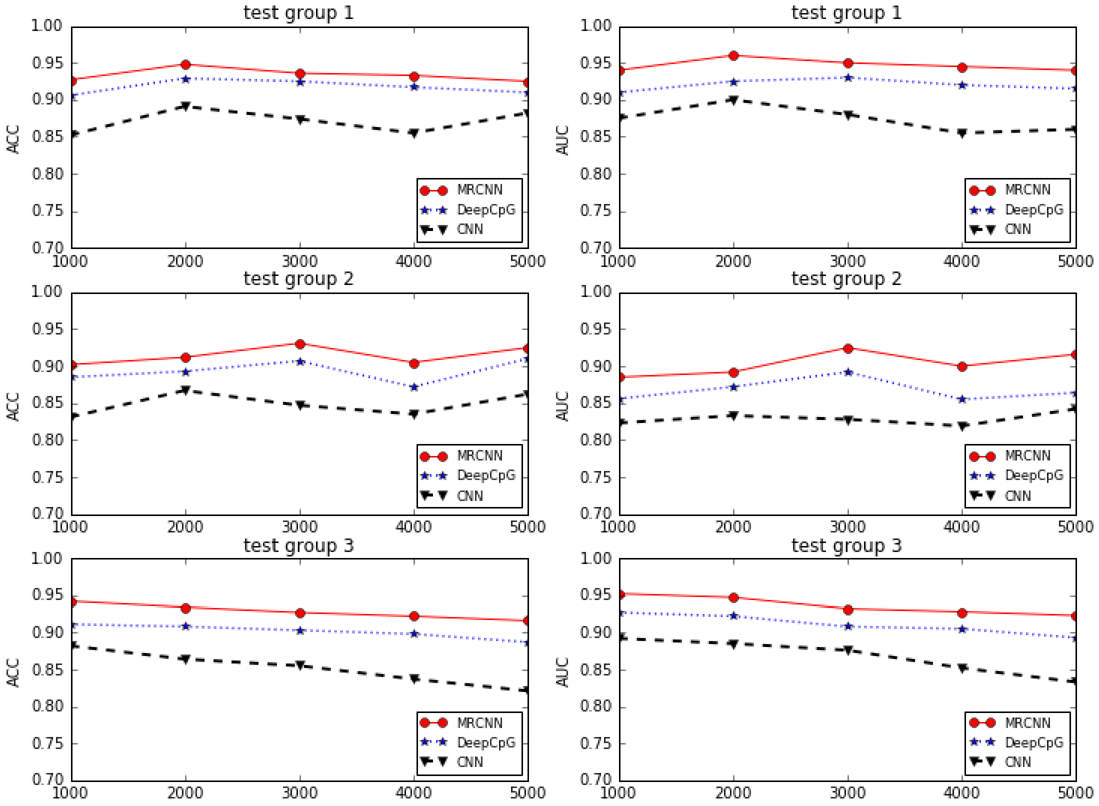

Supplement: Supplementary file 2 — Additional figures. Comparsion of the comprehensive classification performance metrics including ACC and AUC on the different size of test subsets. (PDF 64 kb) [file 12864_2019_5488_MOESM2_ESM.pdf]
