# Supplementary material for: MRCNN: a deep learning model for regression of genome-wide DNA methylation
Source: BMC Genomics. 2019 Apr 4;20(Suppl 2):192. doi: 10.1186/s12864-019-5488-5 (PMC6457069; doi:10.1186/s12864-019-5488-5)

Additional file 3. Comparison of the classification performances in three cancerous tissues.

Among them, Brain_tumor represents human brain astroglioma, Lung_tumor represents H157 non-small cell lung cancer, and Colon_tumor represents colon cancer. MRCNN also achieved good prediction results in the cancer dataset, indicating that the model is also robust in face of complex methylation mechanisms.

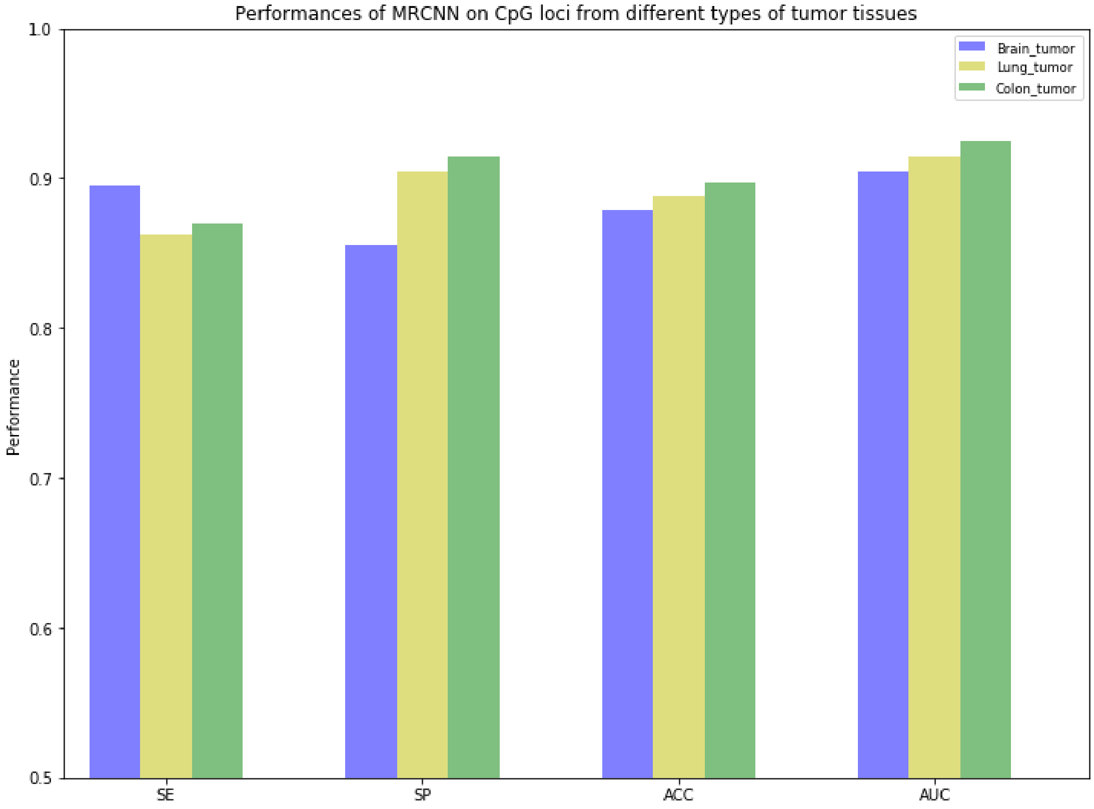

Supplement: Supplementary file 3 — Additional figures. Comparison of the classification performances in three cancerous tissues. (PDF 56 kb) [file 12864_2019_5488_MOESM3_ESM.pdf]
